# The prevalence of thyroid dysfunction in Jordan: a national population-based survey

**DOI:** 10.1186/s12902-022-01166-5

**Published:** 2022-10-20

**Authors:** Kamel M. Ajlouni, Nahla Khawaja, Mohammed EL-Khateeb, Anwar Batieha, Oraib Farahid

**Affiliations:** 1 The National Center (Institute) for Diabetes, Endocrinology and Genetics (NCDEG), University of Jordan, P.O. Box 13165, Amman, 11942 Jordan; 2 Department of Community Medicine, Jordan University of Science and Technology, Irbid, Jordan

**Keywords:** Jordan, Prevalence. Hypothyroidism, Hyperthyroidism

## Abstract

**Background:**

The objectives of this study are to assess the prevalence of clinical and subclinical hypo- and hyperthyroidism and their associated factors among Jordanian adults.

**Methods:**

In a cross-sectional population-based survey, a representative sample that included 3753 Jordanian adults was selected from the 12 governorates that represent the three regions of the country, in the year 2017. Sociodemographic and clinical data were obtained and blood samples were collected from all participants. Thyroid stimulating hormone (TSH), free tri-iodothyronine (FT3), free thyroxine (FT4), thyroglobulin antibody (TgAb) and thyroid peroxidase antibody (TPOAb) were measured to evaluate the thyroid function.

**Results:**

The overall prevalence of thyroid dysfunction was 11.9%. Around 76% of patients with thyroid dysfunction were previously undiagnosed. The prevalence of hypothyroidism and subclinical hypothyroidism was 3.1 and 5.3%, respectively. The prevalence of hyperthyroidism and subclinical hyperthyroidism was 1.0 and 2.5%, respectively. Female preponderance which was mainly related to hypothyroid disorders was evident. The prevalence of positive TPOAb and TgAb in the study population was 14.9 and 15.3%, respectively. The prevalence of detectable TPOAb and TgAb in the euthyroid participants was10.3 and 11.9%, respectively. Logistic regression analysis revealed that female sex, age ≥ 50 years and the presence of TgAb and TPOAb were strongly associated with hypothyroidism. Hyperthyroidism was significantly associated with the presence of TPOAb and age ≥ 50 years.

**Conclusion:**

The prevalence of unrecognized thyroid dysfunction is high among Jordanians. A public health policy of screening high risk groups particularly those ≥50 years of age is recommended.

## Background

The thyroid gland is a crucial part in the human endocrine system as it secretes hormones that regulate development, growth and metabolism [1]. The pleiotropic effects of thyroid hormones on several metabolic pathways are reflected by the diversity of clinical presentations that are commonly age and sex related [1–3]**.** Signs and symptoms of thyroid dysfunction are often vague and nonspecific; thus, the diagnosis of thyroid dysfunction is primarily based on biochemical confirmation.

The spectrum of thyroid dysfunction can extend from subclinical to overt disease that reflects a state of more severe thyroid derangements [2–4]. Commonly, thyroid dysfunction has a substantial impact on the general well-being and is associated with profound morbidity if overlooked or untreated. Hypothyroidism is associated with dementia, hypertension, dyslipidemia, cardiovascular and cerebral events, and if untreated can lead to myxedema coma [2]. Subclinical hypothyroidism is associated with cardiovascular morbidity and mortality and the risk is TSH dependent [5]. Subclinical hyperthyroidism and hyperthyroidism are associated with increased risk of atrial fibrillation, heart failure, dementia, bone loss and fractures [2, 6].

The most common causes of thyroid dysfunction are iodine deficiency in iodine-deficient populations, and thyroid autoimmunity in iodine replete-populations. However, it is worth noting that still one third of the world population is living in iodine deficient areas [7].

Jordan is a small Middle Eastern country, with a population of 10 million [8]. The implementation of Iodine Deficiency Disorders Control Program in 1995 has shifted the status of Jordan to that of a country with adequate iodine intake [9]. According to The National Survey to Assess Iodine Deficiency Disorders (IDD) among school children in Jordan, the minimum urinary iodine concentration was 203 μg/L. Consumption of adequately iodized salt is widespread: 96% of households used adequately iodized salt (≥15 ppm) in 2010 [10]. Previous studies from Jordan had shown that the overall prevalence of thyroid dysfunction in patients with type 2 Diabetes Mellitus and type 1 Diabetes Mellitus was 12.6 and 8.9%, respectively [11, 12]**.** However, these data are not representative of the status of thyroid dysfunction in the general population. Similarly, data on the prevalence of hypothyroidism in the Arab world are limited to studies that included patients who are at high risk for thyroid dysfunction [13]**.**

Although the utility of screening healthy adults for thyroid disorders is debated, studying the epidemiology of thyroid dysfunction in Jordan can be of great benefit in formulating health strategies for the management of this problem and its consequences. The objectives of this study are to determine the national prevalence of hypothyroidism, hyperthyroidism and subclinical thyroid disorders and their associated factors and to assess the prevalence of detactable TPOAb and TgAb among Jordanian adults.

## Methods

The current study is derived from a comprehensive population-based survey that was conducted in Jordan in 2017. The survey was conducted to assess the status of diabetes, hypertension, obesity, dyslipidemia, vitamin D and B12 deficiency, thyroid dysfunction and other aspects of health care for non-communicable diseases (NCDs) in Jordan [14]. The study was approved by the Ethical Committee at the National Center for Diabetes Endocrinology and Genetics (NCDEG), which is accredited by the National Ethics Committee. The study was conducted in accordance with the Declaration of Helsinki. An informed written consent was obtained from each participant. The confidentiality of the information was assured and only used for scientific purposes.

A representative sample of Jordanian adults was selected through a multistage sampling technique from the 12 governorates that represent the three regions of the country, namely, the North, the Middle and the South. The populations of the three regions are similar and homogeneous. In each governorate, 1–3 healthcare centers were initially selected. Then, a team of two field investigators visited a systematic sample of households in the catchment area of the selected health centers and invited all subjects aged ≥18 years to participate in the study, after explaining the study objectives and procedures. Eligible subjects were asked to attend the designated health center in a given day early in the morning, fasting for at least 8 h, and to bring their medications with them. The overall response rate was 78.1%. The total sample participating in the survey was 4056 participants. Subjects with acute illness, chronic diseases as heart failure, renal failure and or hepatic failure and those receiving amiodarone, glucocorticoids, lithium and antiepileptic drugs were excluded from this study. A total of 3753 participants (92.5% of the National study sample) met the inclusion criteria with valid thyroid function tests and were included in the present analyses.

Face to face interviews were conducted by trained interviewers using a predesigned structured questionnaire. Relevant data for the present study included sociodemographic data, data on history of thyroid illness, and medications used. Anthropometric measurements including height and weight were carried out by the field investigators in a standard method. Blood samples were taken by skilled laboratory technicians to measure thyroid stimulating hormone (TSH), free tri-iodothyronine (FT3), free thyroxine (FT4), thyroglobulin antibody (TgAb) and thyroid peroxidase antibody (TPOAb). At the survey site, samples were centrifuged within 1 hour, and transferred by separate labeled tubes in iceboxes to the central laboratory at the NCDEG in Amman, Jordan. All biochemical measurements were carried out by the same team of laboratory technicians using the same method throughout the study period. The normal adult reference range for TSH, FT3, FT4, TPOAb and TgAb used were 0.55–4.78 mIU/l, 3.5–6.5 pmol/ml, 10–22.7 pmol/ml, < 60 U/ml and < 60 U/ml, respectively. Participants who were receiving levothyroxine or carbimazole at the time of the interview were considered to have known hypothyroidism or hyperthyroidism, respectively. Undiagnosed thyroid dysfunction was defined as the participant was not receiving levothyroxine or carbimazole at the time of the interview with abnormal thyroid function tests. Undiagnosed thyroid dysfunction was classified into four categories using the normal reference ranges: primary hypothyroidism (low FT4 combined with high TSH), subclinical hypothyroidism (normal FT4 combined with high TSH), primary hyperthyroidism (high FT4 or high FT3 combined with low TSH) and subclinical hyperthyroidism (normal FT4 and normal FT3 combined with low TSH) [2–4]**.**

TSH was measured by ADVIA Centaur TSH3-Ultra chemiluminescent immunoassay provided by Siemens Diagnostics, Tarrytown, NY. The functional sensitivity of ADVIA Centaur TSH3-Ultra assay is 0.008mIU/l. FT4 and FT3 were measured by ADVIA Centaur FT4 and FT3 assay, respectively using direct chemiluminescent immunoassay provided by Siemens Diagnostics, Tarrytown, NY. TgAb and TPOAb were measured by ADVIA Centaur Anti Tg and Anti TPO assay, respectively using direct chemiluminescent competitive immunoassay provided by Siemens Diagnostics, Tarrytown, NY. The intra-assay coefficients of variation (CV) were: for TSH 1.95%, FT4 2.23%, FT3 3.08%, TPOAb 6.8% and TgAb 5.8%. The inter-assay CV for TSH, FT4, FT3, TPOAb and TgAb were 4.28, 4.0, 4.05%, 3.1 and 2.1%, respectively.

### Data management and statistical analysis

Data were entered and analyzed using the Statistical Package for Social Sciences software (SPSS IBM version 20). Range and logical checks were used to detect data entry errors and corrected accordingly. Continuous variables were presented as means ± standard deviations (SD). To compare between the TSH median groups 2-tailed Mann-Whitney U test was used. Proportions were used to estimate the prevalence of different thyroid disorders. The chi-square test and the independent t test were used to assess the statistical significance of differences in categorical and continuous variables, respectively. Logistic regression was used to assess the independent effects of individual factors associated with hypothyroidism and hyperthyroidism. Also, it was used to assess the independent effects of individual factors associated with antibodies. A *P*- value of less than 0.05 was considered to be statistically significant.

## Results

### Sociodemographic characteristics

This study included 3753 participants (2664 females, 1089 males). The mean age of the study participants was 43.8 years (SD = 14.3) and 85% of them were < 60 years. The mean BMI of the study participants was 29.7 kg/m2 (SD =6.3) and 77% were either overweight or obese. Female and male participants differed significantly in their socio-demographic and anthropometric characteristics as shown in Table 1.

**Table 1:** The socio-demographic and anthropometric characteristics of the study participants according to sex (*n* = 3753)

Variables	All (***n*** = 3753) N (%)	Female (***n*** = 2664) N (%)	Male (***n*** = 1089) N (%)	***P***-value
**Age, mean (SD)**	43.8 (14.3)	42.2 (13.7)	47.8 (14.7)	0.000
18–29	675 (18.0)	535 (20.1)	140 (12.9)	0.000
30–39	755 (20.1)	597 (22.4)	158 (14.5)	
40–49	999 (26.6)	718 (27.0)	281 (25.8)	
50–59	768 (20.5)	505 (18.8)	266 (24.4)	
≥ 60	556 (14.8)	312 (11.7)	244 (22.4)	
BMI (kg/m2), mean (SD)*	29.7 (6.3)	30.1 (6.6)	28.8 (5.5)	0.000
Under weight	86 (2.3)	66 (2.5)	20 (1.8)	0.000
Normal	750 (20.1)	530 (20.0)	220 (20.3)	
Overweight	1211 (32.5)	766 (29.0)	445 (41.1)	
Obese	1682 (45.1)	1283 (48.5)	399 (36.8)	
**Waist circumference (cm)#**				
**Mean (SD)**	94.3 (16.4)	92.5 (16.6)	98.7 (15.1)	0.000
Normal	955 (25.7)	597 (22.6)	358 (33.1)	0.000
Increased	2767 (74.3)	2044 (77.4)	723 (66.9)	
**Geographical region**				
North	1152 (30.8)	817 (30.8)	335 (30.9)	0.001
Middle	1740 (46.6)	1277 (48.2)	463 (42.7)	
South	843 (22.6)	557 (21.0)	286 (26.4)	
**Marital status**				
Married	2856 (76.7)	1914 (72.4)	942 (87.1)	0.000
Single	557 (15.0)	426 (16.1)	131 (12.1)	
Divorced + widow	312 (8.4)	303 (11.5)	9 (0.8)	
**Occupation**				
Unemployed	2374 (65.1)	1932 (75.1)	442 (41.2)	0.000
Employed	1272 (34.9)	641 (24.9)	631 (58.8)	
**Smoking**				
Smoker	526 (14.1)	171 (6.5)	355 (32.9)	0.000
Non-smoker	3192 (85.9)	2468 (93.5)	724 (67.1)	

1. * Normal: 18.5–24.9, over weight: 25–29.9, obese ≥30, and underweight < 18.5

2. #Waist circumference normal values: (M < 94 cm, F < 80 cm), increased values: (M ≥ 94 cm, F ≥ 80 cm

### TSH distribution

In subjects without previously known thyroid dysfunction, the median serum TSH level (2.5th − 97.5th percentile) was 1.8 mIU/l (0.47–7.15 mIU/l). The median serum TSH level in females and males was 1.84 mIU/l (0.47–7.54mIU/l) and 1.68mIU/l (0.47–6.17 mIU/l) (*P* = 0.004), respectively, as s shown in Table 2. Irrespective of sex, there was no age-related increase in the median TSH level. However, a trend towards increasing 97.5th percentiles with age was observed. The 97.5th percentiles in those aged 18–29 years was 6.12 mIU/l compared to 9.72 mIU/l in those aged ≥60 years.

**Table 2:** The median TSH level in participants without known thyroid dysfunction (*n* = 3638) according to Thyroid Antibodies Status^a^

Age	Regardless of Thyroid Antibodies status (***n*** = 3638)		With Negative Thyroid Antibodies (***n*** = 2931)	
	Number	Median TSH (2.5–97.5 centiles)	Number	Median TSH (2.5–97.5 centiles)
**Total population**				
18–29	660	1.82 (0.59–6.12)	562	1.77 (0.61–4.67)
30–39	727	1.82 (0.54–5.49)	600	1.72 (0.54–4.68)
40–49	966	1.76 (0.47–6.49)	772	1.66 (0.49–4.46)
50–59	740	1.84 (0.33–9.27)	557	1.69 (0.43–5.13)
≥ 60	545	1.72 (0.39–9.72)	449	1.59 (0.41–6.78)
All	3638	1.80 (0.47–7.15)	2931	1.69 (0.49–4.87)
***P*-value**		0.316		0.281
**Males**				
18–29	140	1.70 (0.62–4.81)	125	1.65 (0.67–4.61)
30–39	157	1.82 (0.40–5.06)	132	1.79 (0.34–4.91)
40–49	280	1.62 (0.54–6.17)	249	1.54 (0.54–4.44)
50–59	259	1.62 (0.40–8.72)	209	1.57 (0.40–4.44)
≥ 60	242	1.73 (0.32–10.22)	209	1.63 (0.34–6.62)
All	1078	1.68 (0.47–6.17)	936	1.61 (0.48–4.77)
***P*-value**		0.438		0.303
**Females**				
18–29	520	1.85 (0.53–6.21)	437	1.80 (0.53–4.75)
30–39	570	1.82 (0.54–6.03)	468	1.72 (0.55–4.58)
40–49	686	1.83 (0.45–7.51)	523	1.72 (0.47–4.48)
50–59	481	1.95 (0.18–9.77)	336	1.81 (0.40–5.52)
≥ 60	303	1.67 (0.39–9.27)	240	1.56 (0.41–7.41)
All	2560	1.84 (0.47–7.54)	2004	1.73 (0.50–4.96)
***P*-value**		0.178		0.133

^a^Thyroid Antibodies: thyroglobulin antibody (TgAb) and or thyroid peroxidase antibody (TPOAb)

Among those without previously known thyroid dysfunction, 3.3 and 0.8% had TSH level below 0.55 and 0.1 mIU/l, respectively. Also, 6.1% had serum TSH level above 4.78 mIU/l, 4.7% had serum TSH level between 4.79–10 mIU/l and 1.4% had TSH level ≥ 10 mIU/l.

### Prevalence of thyroid dysfunction

Among study participants, 2.9% were known to have thyroid dysfunction. The prevalence of newly discovered primary thyroid dysfunction was 9.0%. As shown in Table 3, the prevalence of hypothyroidism and subclinical hypothyroidism was 3.1 and 5.3%, respectively. On the other hand, the prevalence of hyperthyroidism and subclinical hyperthyroidism was 1.0 and 2.5%, respectively. Among participants without previously known thyroid dysfunction, thyroid function test analysis showed a picture suggestive of unclassified thyroid disorders in 33 subjects (0.9%) and a picture suggestive of central hypothyroidism in 15 subjects (0.4%).

**Table 3:** The Prevalence of thyroid dysfunctions among study participants according to sex (n = 3753)

***Thyroid Dysfunction***	***All (N = 3753)*** ***N (%)***	***Females*** ***(N = 2664)*** ***N (%)***	***Males*** ***(N = 1089)*** ***N (%)***	***P-value***
**Newly discovered primary Hypothyroidism**	24 (0.6%)	20 (0.8%)	4 (0.4%)	0.181
**Known hypothyroidism**	95 (2.5%)	89 (3.3%)	6 (0.6%)	0.000
**Subclinical hypothyroidism**	198 (5.3%)	160 (6.2%)	38 (3.5%)	0.002
**Newly discovered Hyperthyroidism**	23 (0.6%)	17 (0.7%)	6 (0.6%)	0.756
**Known hyperthyroidism**	15 (0.4%)	10 (0.4%)	5 (0.5%)	0.857
**Subclinical hyperthyroidism**	95 (2.5%)	68 (2.7%)	27 (2.5%)	0.89

The prevalence of hypothyroidism in females, was 4.1% in comparison to 0.9% in males (*P* = 0.000). The prevalence of subclinical hypothyroidism in females and males was 6.0 and 3.5%, respectively (*P* = 0.002). However, no sex related differences in the prevalence of hyperthyroid disorders was detected.

**Fig. 1:**
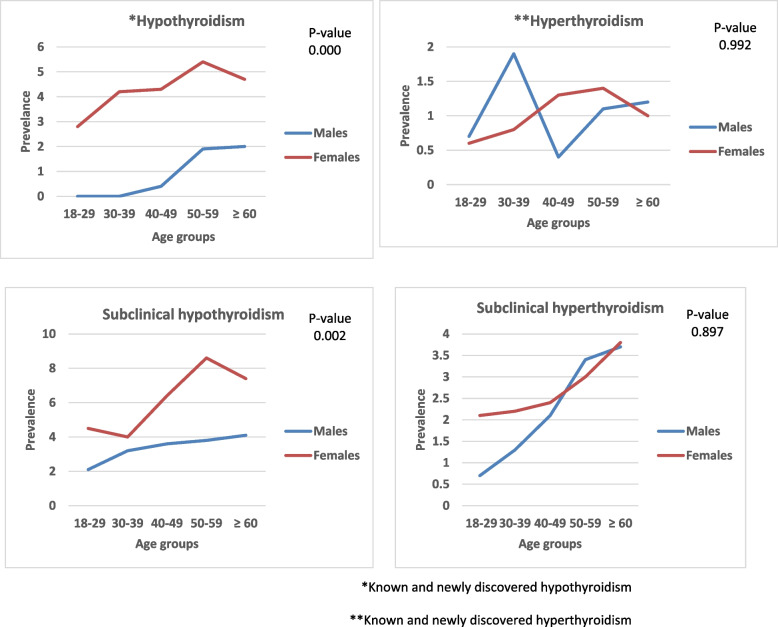
The prevalence of different types of thyroid dysfunction according to age and sex among study participants (*n* = 3753). *Known and newly discovered hypothyroidism. **Known and newly discovered hyperthyroidism

In females, the prevalence of of different types of thyroid dysfunction increased with age as shown in Fig. 1. The highest prevalence of hypothyroidism, subclinical hypothyroidism and hyperthyroidism was among females aged 50–59 years (5.4%,8.6 and 1.4% respectively), whereas, the highest prevalence of subclinical hyperthyroidism was in females older than 60 years (3.8%). In males, a trend towards an increase in the prevalence with age was noticed for hypothyroidism and subclinical thyroid dysfunction as shown in Fig. 1.

### Factors associated with hypothyroidism and hyperthyroidism

Logistic regression was used to assess the independent effects of individual factors associated with total hypothyroidism and total hyperthyroidism. Total hypothyroidism includes all types of hypothyroid dysfunction (known hypothyroidism, newly discovered hypothyroidism and subclinical hypothyroidism). Total hyperthyroidism includes all types of hyperthyroid dysfunction (known hyperthyroidism, newly discovered hyperthyroidism and subclinical hyperthyroidism).

Females were 1.86 times more likely to have hypothyroidism than males (95% CI = 1.32–2.60, *P* = 0.000). The likelihood of hypothyroidism was higher among participants in 50–59 and ≥ 60 years age groups (OR = 1.65 and 1.64, *P* = 0.018 and 0.039, respectively). The presence of TPOAb was the strongest predictor for hypothyroidism; compared to participants with negative antibodies, those with positive TPOAb were 7.42 times more likely to have hypothyroidism (95% CI = 5.40–10.20, *P* = 0.000). In addition, the presence of TgAb was associated with increased odds for hypothyroidism as shown in Table 4.

**Table 4:** Variables associated with thyroid dysfunction in multivariate logistic regression analysis

Variables	^a^Total hypothyroidism		^b^Total hyperthyroidism	
	OR (CI)	***P***-value	OR (CI)	***P***-value
**Sex**				
Male	1	0.000	1	0.757
Female	1.86 (1.32–2.60)		1.06 (0.72–1.58)	
**Age (years)**				
18–29	1		1	
30–39	1.13 (0.72–1.77)	0.597	1.29 (0.68–2.48)	0.430
40–49	1.34 (0.88–2.02)	0.164	1.38 (0.75–2.54)	0.293
50–59	1.65 (1.08–2.51)	0.018	1.85 (1.01–3.40)	0.047
≥ 60	1.64 (1.03–2.62)	0.039	2.12 (1.12–4.01)	0.020
**Thyroid peroxidase antibody (TPOAb)**				
Negative	1	0.000	1	0.014
positive	7.42 (5.40–10.20)		1.93 (1.14–3.27)	
**Thyroglobulin antibody (TgAb)**				
Negative	1	0.000	1	0.327
positive	1.98 (1.44–2.74)		0.75 (0.42–1.33)	

^a^Total hypothyroidism = Known hypothyroidism + subclinical hypothyroidism + newly discovered hypothyroidism

^b^Total hyperthyroidism = Known hyperthyroidism + subclinical hyperthyroidism + newly discovered hyperthyroidism

On the other hand, the likelihood of hyperthyroidism was higher among participants with detected TPOAb (OR = 1.93, 95% CI = 1.14–3.27, *P* = 0.014). Compared to participants in the third decade of life, those aged 50–59 and ≥ 60 were 1.85 times and 2.12 times, respectively, more likely to have hyperthyroidism (*P* = 0.047 and 0.020, respectively).

### Thyroid antibodies

The prevalence of positive TPOAb and TgAb in the study population was 14.9 and 15.3%, respectively. The proportion of participants who had detected TPOAb was 10.3% in the euthyroid group, 65% in hypothyroidism group, 57.6% in subclinical hypothyroidism group, 47.4% in hyperthyroidism group and 12.6% in subclinical hyperthyroidism group. The proportion of participants who had detected TgAb was 11.9% in the euthyroid group, 52.1% in hypothyroidism group, 49% in subclinical hypothyroidism group, 31.6% in hyperthyroidism group and 11.6% in subclinical hyperthyroidism group. The percentage of total hypothyroidism patients with negative both TPOAb and TgAb was 12.7%.

**Fig. 2:**
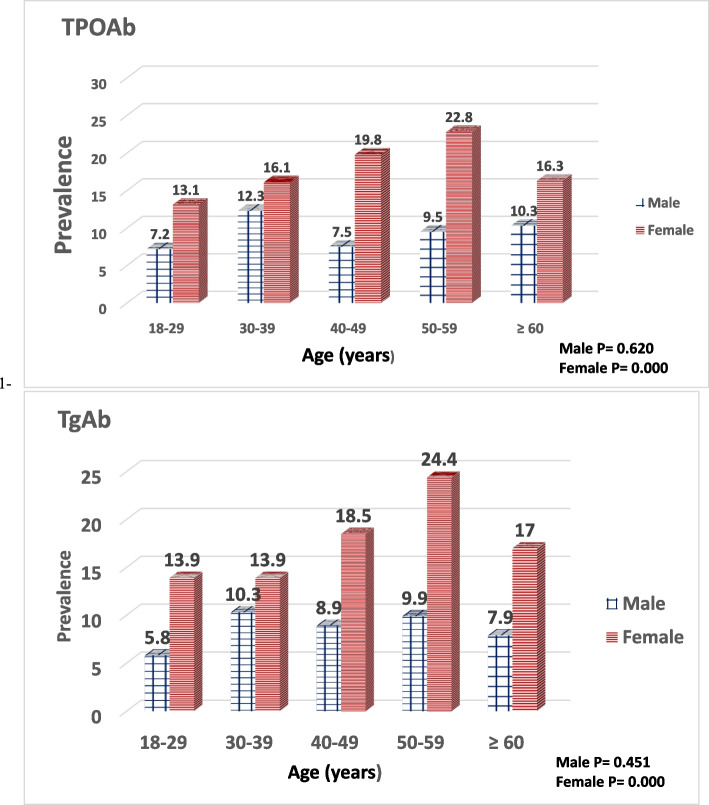
The prevalence of positive TPOAb and TgAb in the study population according to age and sex (*n* = 3753)

The prevalence of positive TPOAb in males and females was 8.3 and 15.7%, respectively (*P* = 0.000). The prevalence of positive TgAb in males and females was 9.3 and 17.8%, respectively (*P* = 0.000). Age-related increase in the prevalence of detected TPOAb or TgAb was observed in females as shown in Fig. 2.

Logistic regression was used to assess the independent effects of individual factors associated with TPOAb and TgAb. Females were twice as likely to have TPOAb than males (95% CI = 1.55–2.59, *P* = 0.000). Also, Females were 1.91 times more likely to have TgAb than males (95% CI = 1.50–2.42, *P* = 0.000). Subjects in their 6th decade of life were 1.79 and 1.61 times more likely to have TPOAb and TgAb, respectively than those aged 18–29 years (*P* = 0.001 and 0.003, respectively). The odds ratio for having TPOAb in total hypothyroidism was 11.88 (95% CI = 9.2–15.34, *P* = 0.000). The odds ratio for having TPOAb in total hyperthyroidism was 2.46 (95% CI = 1.61–3.77, *P* = 0.000). Subjects in the total hypothyroidism group were 6.68 times more likely to have TgAb (95% CI = 5.23–8.55, *P* = 0.000).

## Discussion

This study evaluated the prevalence of thyroid dysfunction among adult Jordanians at the national level. The overall prevalence of thyroid dysfunction in this population was 11.9%. The prevalence of primary hypothyroidism and hyperthyroidism was 3.1 and 1.0%, respectively. The prevalence of subclinical thyroid dysfunction was 7.8%.

In the current study, a trend toward age-related increase in the median TSH level was not observed. However, the trend toward increasing 97.5 centiles with age irrespective of the sex supports the population shift toward higher TSH concentrations with aging observed in the NHANES III study [15]. It has been hypothesized that the shift may be part of a healthy aging process due to altered sensitivity of TSH receptors or hypothalamic pituitary feedback system, or change in the biological activity of TSH molecule [15, 16]**.** It is noteworthy that the population of Jordan is relatively young as only 3.7% are older than 65 years [8]**.** Nevertheless, a large-scale study that includes a larger proportion of elderly is needed to set a reference range for TSH level.

The prevalence of elevated TSH (> 4.78 mIU/l) in our study was 6.1%, while, the prevalence of elevated TSH in Netherlands, Japan, The Health Study of Nord-Trondelag (HUNT), Australia, Colorado, Rotterdam and the Framingham studies was 4.4, 4.5, 5.3, 5.6, 9.5, 11, and 13%, respectively [17–23]**.** The prevalence of suppressed TSH (< 0.55 mIU/l) in the current study was 3.3%, while, the prevalence of suppressed TSH in Netherlands, Japan, The Health Study of Nord-Trondelag (HUNT)**,** Australia and Colorado study was 1.2, 0.9, 0.6,0.64 and 2.2%, respectively [17–21]. This discrepancy may be related to the differences in the study population, definition of the disease state, iodine intake and laboratory assay differences.

In this study the overall prevalence of hypothyroidism was 3.1%. Our finding is comparable to that of a recent meta-analysis which included 14 studies from Europe, in which the prevalence of hypothyroidism was 3.05% [24]**.** This finding is inconsistent with studies from areas with sufficient iodine intake. The prevalence of hypothyroidism in areas of sufficient iodine intake as in Tehran thyroid study, Colorado study, Spain and NHANES III study was 2, 0.4 0.3 and 0.3% respectively [20, 24–26].**.**On the other hand, the prevalence of hyperthyroidism among this study cohort is comparable to that reported from Spain which was 0.8% [26]. But, it is lower than that previously reported from several studies. For instance, the prevalence of hyperthyroidism in Tehran thyroid study, Europe, NHANES III and Colorado study was 2.2, 0.75, 0.5 and 0.1% respectively [21, 24, 25, 27]. A previously reported study from Jordan had shown a significantly higher prevalence of hypothyroidism when compared to the international studies that reached 9.4% and a lower prevalence of hyperthyroidism which reached 0.58% [28]. The inconsistency in reported prevalence of different types of thyroid dysfunction could be related to the differences in study design, racial and genetic background, age, environmental factors, iodine consumption, criteria used to define different types of thyroid dysfunction and the laboratory assay sensitivities used to measure thyroid hormones levels [24].

The majority of thyroid dysfunction in the current study were undiagnosed and 86% of them were subclinical. Similarly, subclinical thyroid disorders represented 80, 85, 88 and 90% of the undiagnosed thyroid dysfunction in Europe, Spain, NHANES III and Colorado study, respectively [21, 24, 26, 27]. In the same context, the past two decades have witnessed a rise in the prevalence of subclinical thyroid dysfunction. This rise is related to the substantial increase in thyroid function testing with ultra-sensitive assays and the decline in TSH thresholds for levothyroxine initiation [29]**.**

Sex distribution for thyroid dysfunction in this study clearly demonstrated a female predominance, which was mainly driven by hypothyroidism and subclinical hypothyroidism. The females’ higher prevalence of detected thyroid autoantibodies, in the background of sufficient iodine intake in Jordan could account for this female predilection [27, 30].

In iodine replete-populations, the most common cause of thyroid dysfunction is thyroid autoimmunity. The presence of thyroid autoantibodies particularly TPOAb are considered as a surrogate marker for future development of thyroid dysfunction [31]**.** On the other hand, thyroid autoantibodies could be present in normal population, but the likelihood is significantly higher in subjects with thyroid dysfunction [27]**.** Among our study participants, the prevalence of detectable TPOAb and TgAb in the euthyroid participants was10.3 and 11.9%, respectively. Similarly, in NHANES III study, 12 and 10% of the healthy population had detectable TPOAb and TgAb [27].

This study has many strengths. This study is one of the few studies in the region that assessed the prevalence of thyroid dysfunction and their correlation with thyroid antibodies in the general population using a large representative sample. It provides a better understanding for the distribution of thyroid disease patterns in Jordan and sheds light on the prevalence of unknown thyroid dysfunction. The use of highly sensitive laboratory assays further enhances the validity of the current study results. Finally, determination of a Jordanian specific reference range for TSH level will aid in the future classification and management of thyroid dysfunction.

However, this study has several limitations. First, the female response rate was significantly higher than males. This has been consistently observed in a series of surveys previously conducted in Jordan. The main reason for this finding is the higher employment rate in males which makes their participation more difficult. Second, urinary iodine was not measured, which is needed to assess iodine intake. Finally, the study is cross-sectional in nature and, thus, observed associations can’t be considered as causal because of the possibility of survival and temporal biases associated with this study design.

## Conclusion

The prevalence of unrecognized thyroid dysfunction is high among Jordanians. As the majority of the current study population are young with a long life expectancy, the likelihood of developing long term complications related to thyroid dysfunction is high, if missed or left untreated. Therefore, a public health policy of screening high risk groups particularly females and those ≥50 years of age permits early diagnosis and appropriate timely treatment of comorbidities related to thyroid dysfunction. Also, a large-scale study that includes a larger proportion of elderly is recommended to set a reference range for TSH level, to avoid unnecessary levothyroxine treatment.

## Data Availability

The datasets used and analyzed during the current study are available from the corresponding author on reasonable request.

## Abbreviations

TSH: Thyroid stimulating hormone
FT3: Free tri-iodothyronine
FT4: Free thyroxine
TgAb: Thyroglobulin antibody
TPOAb: Thyroid peroxidase antibody
IDD: Iodine Deficiency Disorders
NCDs: Non-communicable diseases
NCDEG: National Center for Diabetes Endocrinology and Genetics
SD: Standard Deviations
CI: Confidence Intervals
SPSS: Statistical Package for Social Sciences software
NHANES: National Health and Nutrition Examination Survey
HUNT: The Health Study of Nord-Trondelag
